# The voting experience and beliefs about ballot secrecy

**DOI:** 10.1371/journal.pone.0209765

**Published:** 2019-01-07

**Authors:** Conor M. Dowling, David Doherty, Seth J. Hill, Alan S. Gerber, Gregory A. Huber

**Affiliations:** 1 Department of Political Science, University of Mississippi, Oxford, MS, United States of America; 2 Department of Political Science, Loyola University Chicago, Chicago, IL, United States of America; 3 Department of Political Science, University of California, San Diego, La Jolla, CA, United States of America; 4 Department of Political Science, Yale University, New Haven, CT, United States of America; Augusta University, UNITED STATES

## Abstract

New democracies go to great lengths to implement institutional protections of the electoral process. However, in this paper we present evidence that shows that even in the United States—where the secret ballot has been in place for generations—doubts about the secrecy of the voting process are surprisingly prevalent. Many say that their cast ballot can be matched to their name or that others could observe their vote choices while they were voting. We find that people who have not previously voted are particularly likely to harbor doubts about the secrecy of voters’ ballots. Those who vote by mail in the privacy of their own homes also feel that others are able to discover their vote choices. Taken together, these findings suggest an important divergence between public perceptions about and the institutional status of the secret ballot in the United States, a divergence that may affect patterns of voting behavior and political participation.

## Democratic citizenship

Democracies are often defined by the rules and procedures that govern their elections. Great effort is put forth to create a system of laws and procedures that promote “fair elections” where the outcome is likely to be widely viewed as legitimate and losing factions accept defeat in peace. Many of these rules and procedures are designed so that all citizens can make choices on their ballots as they see fit and participate in the political process without fear of harassment from employers, neighbors, and elected officials. However, formal rules and institutions may only have their intended effect if people understand how they are structured and believe that they work.

We investigate whether perceptions of the operation of a key democratic institution, the secret ballot, deviate from the legal and practical reality of that institution. Such deviations may be consequential. Lack of information (or misinformation) about the secrecy of the voting process may affect who votes and, perhaps, which candidates voters support on Election Day [1–3]. More broadly, doubts about ballot secrecy or the functioning of other institutions—even institutions that objectively appear to work quite well—may foster a sense that the state lacks legitimacy. This may also lead to public demands for unnecessary, costly, or counter-productive changes to institutional rules or procedures.

Prior work finds that many people report general doubts about the secrecy of the voting process and that these doubts may have repercussions for voting behavior [2]. For example, Karpowitz et al. [3] find that the design of polling places can affect perceptions of secrecy, which may in turn affect voters’ behavior—especially the behavior of voters who believe they hold views that conflict with others in their community. Other work finds that these doubts can be partially remediated through the provision of factual information about the voting process [1]. Overall, although we know that many Americans harbor doubts about the secrecy of the ballot, little is known about exactly how people believe the secrecy of the ballot might be compromised. What facets of the voting process lead people to think the process of casting a ballot is partially a public, rather than private act?

We conducted a national survey to gather fine-grained measures of citizens’ experiences and perceptions about aspects of the voting process that might lead to doubts about ballot secrecy. The survey allows us to shed light on exactly how people think their vote choice might be discovered by others. We consider elements of the voting process tied to the operation of formal institutions (e.g., do people think the ballots they cast include personally identifying information?), as well as less formal, social aspects of the process (e.g., do people think other voters will ask about or can see their choices?). Improving our understanding of the contours of doubts about ballot secrecy is likely to be important to practitioners who wish to develop more effective interventions to ensure that the act of voting is *experienced* as private. We make two contributions to understanding of public perceptions about the secrecy of the voting process.

First, we present detailed evidence about the prevalence of doubts about ballot secrecy, as well as about perceptions about how secrecy may be compromised by election officials or other voters—perceptions that may lead people to view voting as a public, rather than private act. We find that although most citizens in the contemporary United States believe their voting choices are secret, a substantial number of citizens harbor doubts or misunderstandings about the voting process. Many believe that their ballot includes personally identifying information, that elected officials can access records of their vote choices, or that other voters at the polling place will observe or ask about their choices.

Second, we assess whether these perceptions vary with key features of individuals’ voting experiences. We examine differences between those who cast paper versus electronic ballots and between early voters and those who voted on Election Day. Although these may be different populations (that is, the types of people choosing different discretionary options may be different), these comparisons suggest that those who cast paper ballots in-person on Election Day harbor fewer doubts about ballot secrecy than those who cast electronic or early ballots. However, with some notable exceptions, the magnitudes of the differences we find are modest. We do find evidence that those who vote by mail report experiences and perceptions that are inconsistent with the notion that voting is a private act. We also compare the perceptions of respondents who reported voting in the last general federal election to those who said they either had never voted or had voted, but skipped the last election. These comparisons reveal substantial differences. Those who indicated that they did not participate in the previous election are less certain about the secrecy of the process, and those who say they have never voted are even more likely to harbor doubts. For example, compared to those who have voted, non-voters are much less likely to say that voters do not write their name on their ballot.

In the next section of the paper we briefly outline prior work on the importance of the secret ballot and citizen beliefs about its proper implementation. We also briefly lay out reasons to expect these perceptions to vary with voting technology and timing (early v. Election Day), as well as with how recently one has voted—or whether one has ever voted. We then describe our data source, the basic characteristics of our sample, and respondents’ general beliefs about the secrecy of their vote choices. Next, we describe the voting experiences and perceptions of respondents who reported casting an in-person paper or electronic ballot—either early or on Election Day—the last time they voted. Then, we assess how these perceptions vary with voting technology (with a particular eye to the experiences of voters who cast their ballots by mail), timing of voting (early versus on Election Day), and between recent voters and those who have either not voted recently or have never voted.

## Beliefs about Ballot Secrecy

The secret ballot is one of a set of democratic institutions—e.g., freedom of speech, freedom of association (allowing competing political parties), universal suffrage, and due process of law—designed to foster competitive and legitimate democratic elections. The ability to vote without one’s choices being revealed to others is considered an essential characteristic of legitimate democratic systems [4]. The secret ballot helps protect voters from fear of intimidation or coercion. In the United States, for example, a secret (Australian) ballot was adopted beginning in the late 1800s in an effort to transform the process of voting from a public affair that was improperly influenced through bribery, coercion, and intimidation to a more private affair free of such maladies [5–8]. Since its adoption, with the exception of some party nominating caucuses, the secret ballot has been used in all federal and state elections of government officials. As such, it is generally assumed that voting in the U.S. occurs in secret; that is, voting is a private act and vote choices are not divulged absent outright fraud unless an individual voluntarily reveals her own choices to others. However, recent evidence demonstrates that in a world of imperfect human implementation and uneven knowledge, citizen perceptions of democratic institutions do not precisely mirror the legal status of those institutions. Specifically, previous work finds that a surprising number of Americans harbor doubts about the secrecy of their ballots [2].

Other work suggests that perceptions about ballot secrecy are likely to be consequential. Individuals’ experiences at the polling place, including their sense of privacy while voting, can affect both evaluations of poll workers and confidence in the electoral process as a whole [9]. In a field experiment, Karpowitz et al. [3] show that polling place procedures designed to increase the privacy of voting can increase perceptions of secrecy. They also find that most of the effect of the treatment occurs for voters in the local political minority, who may otherwise be more concerned that their choices would be revealed to members of the majority. Stewart, Alvarez, and Hall [10] find that greater concern about the privacy of the vote is associated with less confidence that the vote is accurately counted.

Two aspects of the process of casting a ballot may be particularly important for understanding people’s voting experiences and the sources of doubts about the secrecy of one’s ballot. First, the official formal aspects of the voting process, including interactions with election administrators and perceptions about whether voting procedures protect the anonymity of their ballots, may play an important role in whether people think of voting as a private or public act. Although voting in the United States is conducted by secret ballot and is explicitly designed to ensure the privacy of people’s choices on Election Day, many people may be unfamiliar with the rules and regulations election administrators must adhere to and may therefore think that their vote choices are a matter of public record. Alternatively, people may not be confident that these procedures work.

Second, the social aspects of the voting process may affect how people think about voting. These social aspects include people’s assessments of whether their polling place provides an environment where they can cast their ballots privately, as well as whether people go to the polls alone or with other voters and whether they discuss their choices with others. Even if people recognize formal ballot secrecy protections and trust election officials, they may nonetheless view voting as a public act. For example, they may assume that other voters can see their ballots while they are filling them out or that other voters will ask them to discuss their choices at the polling place.

Beyond offering new evidence regarding aggregate perceptions about ballot secrecy, we consider several key aspects of citizens’ voting experience that may be tied to perceptions about ballot secrecy. First, we compare the perceptions of in-person voters who cast paper ballots to those who cast electronic ballots. Although ballots recorded electronically are, in theory, no different in terms of the level of anonymity they provide, existing work suggests that people harbor doubts about electronic voting [11–12]. Specifically, this work finds that those who cast electronic ballots tend to be less confident that their votes are counted properly. There is also evidence that a non-trivial share of the public believes electronic voting technology is susceptible to fraud [13].

Second, we consider whether secrecy perceptions vary depending on when and where individuals cast their ballots. We compare the perceptions and experiences of those who voted in-person on Election Day to those who cast early, in-person ballots. We posit that those who vote early are less likely to face long lines and busy polling places and, on average, may therefore feel that their ballot is easier to identify because it is not being immediately pooled with as many other ballots. It is also possible that early voters harbor a misperception that election officials need to be able to personally identify their early ballot to ensure that they do not vote more than once.

We also assess whether those who reported voting by mail diverge in their perceptions about the secrecy of their ballots. As with electronic voting, existing work finds that those who cast mail ballots are less confident that their votes will be counted properly [13] and may have doubts about the effectiveness of precautions taken to ensure the anonymity of a ballot that was sent to them personally at their home. An additional possibility that we investigate below, is that, in contrast to casting a vote in a polling place, those who complete their ballot at home are unlikely to do so behind a privacy screen. Indeed, the evidence we report below suggests that many voters who cast mail ballots fill out their ballot in the presence of others.

Finally, we compare the secrecy perceptions of those who reported voting in the federal election prior to our survey to those for whom the experience of voting may be more remote. In particular we compare the reported experiences and perceptions of these individuals to those reported by individuals who said they had voted before but did not vote in the most recent election. We also compare the beliefs of recent voters to the secrecy expectations reported by individuals who said they had never voted before. Again, there is reason to expect differences across these groups. Casting a secret ballot is an unusual act compared to most other forms of human interaction. Typically, when we are called upon to make a choice we are expected to disclose our preferences directly. Thus, memories of one’s last voting experience may become murky quite rapidly. In contrast to those who cast a ballot a month prior (our survey was fielded about a month after the election), those who have not voted in two years may fail to recollect important details about the voting experiences and report greater doubts about the privacy the process affords. Similarly, those who have never experienced voting may harbor even greater doubts because they have not directly experienced the voting process.

## Measuring perceptions of the voting experience

To investigate these questions, we fielded a survey in December of 2010 through YouGov/Polimetrix, which uses a combination of sampling and matching techniques to approximate a random digit dialing sample. The Yale University Institutional Review Board approved this study as exempt. Section A of the S1 Appendix contains more information on YouGov’s sampling methodology and Section B includes complete question wording for each item discussed in the text. The final weighted sample is nationally representative of the U.S. adult population (25 and over). Unless otherwise noted, all descriptive statistics and analyses presented below use the sampling weights provided by YouGov/Polimetrix. Replication materials are available via the Harvard Dataverse, DOI: https://doi.org/10.7910/DVN/ZDSD9S.

Our core survey questions focused on respondents’ experiences in the election in which they had most recently voted. We exclude 124 respondents (out of a total of 3,000 respondents who began the survey) from the analysis we present below. These respondents include those who: did not indicate when they last voted (N = 4); reported voting but did not recall when or how they cast their ballot (N = 17); and reported having voted in-person on Election Day, but either cast “lever” ballots (N = 72) or an unidentified technology (N = 31). Thus, our full sample consists of 2,876 observations. We present the unweighted descriptive characteristics of our sample in Table S1 of the Supplementary information document.

Approximately 82 percent of respondents in our sample reported voting in the 2010 general election, five percent reported last voting between the 2008 and 2010 general election, six percent said they last voted in the 2008 general election, and three percent said the last time they voted was prior to the 2008 election. One hundred and twenty-one (unweighted) respondents—4.6 percent of the weighted sample—reported never having voted. The reported rate of turnout—82 percent—is high, perhaps because participants in opt-in surveys tend to be more interested or informed than an average citizen [14]. We were able to match 82 percent of our sample (2,368 respondents) to validated voter data gathered as part of the 2010 CCES. Consistent with this explanation, among the 90 percent of these respondents for whom the validation process was successful (our content was fielded before the CCES sample was finalized, thus 18% of our respondents cannot be matched to the finalized, validated 2010 CCES data set), the vast majority (86 percent) of those who said they voted in 2010 are validated as having done so.

Consistent with previous research that has examined whether people harbor doubts about the basic secrecy of the voting process, we find that a substantial proportion of respondents express concerns about the secrecy of their vote choices. We asked, “When you cast a ballot, are your candidate and other vote choices kept secret unless you tell someone, or might your ballot be revealed to others or matched to your name without your permission?” Approximately 12 percent of the weighted sample said their choices were not kept secret. A second question asked how easy respondents thought it would be for others to discover their choices, regardless of whether they believed their choices were generally kept secret. Specifically, we asked, “According to the law, which candidate you vote for is supposed to be kept secret unless you tell someone. Even so, how difficult do you think it would be for politicians, union officials, or the people you work for to find out who you voted for, even if you told no one?” Thirty-six percent of respondents reported that it would be either “not difficult at all” (12 percent) or “not too difficult” (24 percent). Note that seven respondents did not provide responses to each of these items, thus N = 2,869. This compares to 37 percent, 40 percent, and 41 percent in opinion surveys fielded in 2005, 2008, and 2010, respectively [2].

### Perceived limitations of the secrecy of in-person ballots

Among respondents who reported having voted before, 67 percent said that the last time they voted they did so in person on Election Day and an additional 14 percent reported voting early in person. Nineteen percent reported voting via mail or absentee ballot (less than one percent said they did not know how they had voted and are excluded from the present analysis)—a distribution that is consistent with previous surveys [15]. We asked the 2,207 respondents who indicated that they had last voted by casting an in-person ballot (either early or on Election Day) a number of questions about their voting experience. Four of these questions pertain to ways ballot secrecy could be compromised by election officials and four pertained to perceptions regarding whether one’s choices might be discovered by other voters. We begin by considering the first type of challenge to ballot secrecy.

**Fig 1:**
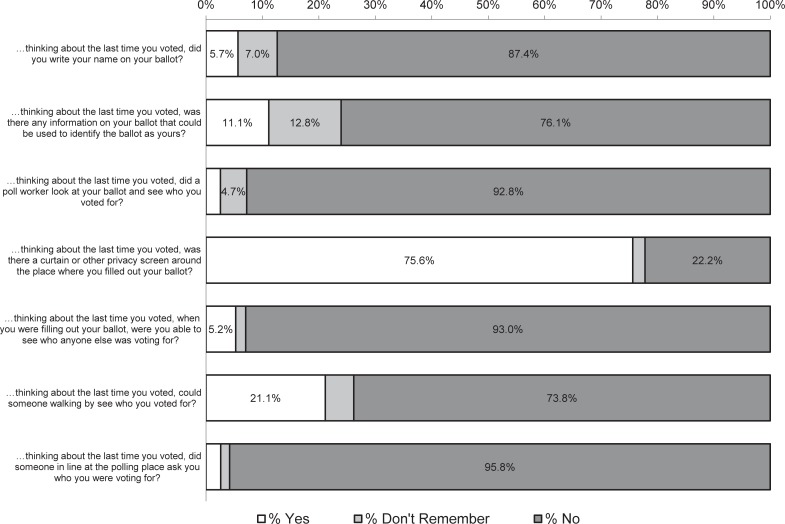
The voting experience of in-person voters. Note: Bar entries are weighted percentages. Empty bars are 2.6% Yes to "…did a poll worker look at your ballot …?", 2.2% Don't Remember to "…was there a curtain or other privacy screen …?", 1.8% Don't Remember to " …were you able to see who anyone else was voting for?", and 2.6% Yes and 1.6% Don't Remember to " …did someone in line at the polling place ask you who you were voting for?". Question on elected officials accessing voting records was asked of 40 percent of respondents. N ranges from 2,197 to 2,205.

We asked a random subset of respondents (N = 889) whether they thought elected officials can access their voting records to figure out who they had voted for and found that 36 percent of in-person voters answered “Yes.” In short, a substantial share of voters cast ballots that they believe can be accessed by officials. We present responses to the remaining three questions about formal threats to ballot secrecy, as well as the four questions about disclosure to other voters in Fig 1. The figure displays the proportion of respondents (weighted to reflect a national sample) who responded “Yes” (white part of the bars), “Don’t Remember” (light gray part of the bars), and “No” (dark gray part of the bars) to each statement. We present the full question wording in the left-hand column. For each item, “Yes” implies an affirmative indication that the voting process lacked privacy. We note that “Don’t Remember” responses do not affirm respondent confidence in the anonymity or privacy of the voting process. Instead these responses suggest uncertainty about the secrecy of the voting process.

As the figure shows, for beliefs about the anonymity of their physical ballots, a non-trivial proportion of respondents reported either writing their name on their ballot (six percent) or that there was some information on their ballot that could be used to identify the ballot as theirs (11 percent). Respondents who said that their ballot contained personally identifying information were asked what that information was in an open-ended follow-up question. Fifty percent said that their ballot listed an identifying code (e.g., a voter identification or social security number), 30 percent said their name or information about their address was listed. Approximately 12 percent indicated that they did not know what the identifying information was and the remaining eight percent provided responses that were too vague to code (e.g., “sticker”). Additionally, over seven percent of respondents said they did not remember whether they had written their name on their ballot and 13 percent said they did not remember if their ballot included identifying information. In other words, our findings indicate that, for example, nearly 25 percent of in-person voters did not affirm that the ballot they cast was devoid of personally identifying information. The final question asked whether a poll worked looked at the respondent’s ballot. Only three percent of in-person voters said that a poll worker looked at their ballot and saw who they were voting for, with an additional five percent saying they did not remember.

The bottom four items asked in-person voters about whether their choices might have been revealed to other voters during the process of voting. Only three percent said someone at the polling place asked them who they were voting for and only five percent said that they were able to see who another voter was voting for. However, 22 percent said there was no curtain or privacy screen around their voting space and over 20 percent said that someone walking by could see who they were voting for.

Note that the values reported in the figure, along with sample sizes for each item (which range from 2,197 to 2,205) are presented in tabular form in the S2 Table of the Supplementary information document. We also report parallel analysis limited to individuals who were validated as having voted in the 2010 general election in that table. The differences we identify using validated turnout are consistent with patterns we report below—those who cast ballots in the most recent election were modestly less likely to report doubts about the secrecy of the voting process.

Taken together, the findings reported in Fig 1 reveal that aspects of the voting process may lead people to view their ballot as something other than strictly anonymous. For example, the fact that almost 25 percent of respondents were not sure whether their ballot was free of personally identifying information suggests that a surprisingly large number of in-person voters are either unsure about whether the ballots they cast can be identified as theirs or affirmatively believe that they can. Similarly, many believe that elected officials can access information about who they voted for. Additionally, many in-person voters reported that there was ample opportunity for someone walking by them to see who they voted for. In short, in spite of the fact that ballot secrecy is a long-standing feature of the American political landscape, many American voters are either not aware of, or harbor doubts about, the implementation of the secret ballot.

## Variation in secrecy perceptions across voting methods and timing

**Fig 2:**
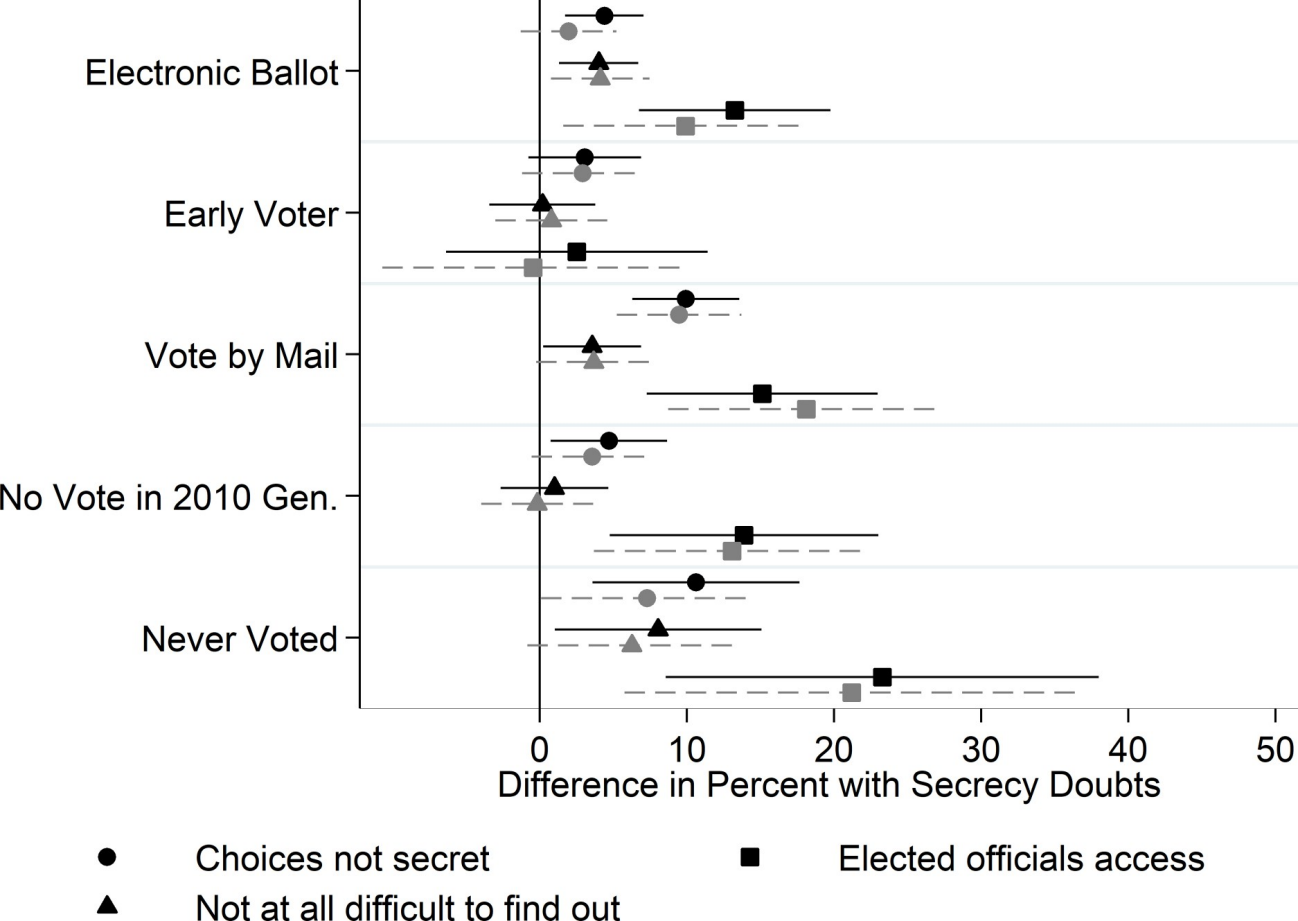
Top-level secrecy perceptions. Whiskers indicate 95% confidence intervals. Gray markers are from models that control for respondent demographics. Reference categories: Paper ballot; Election Day voter; Voted in 2010 General election.

**Fig 3:**
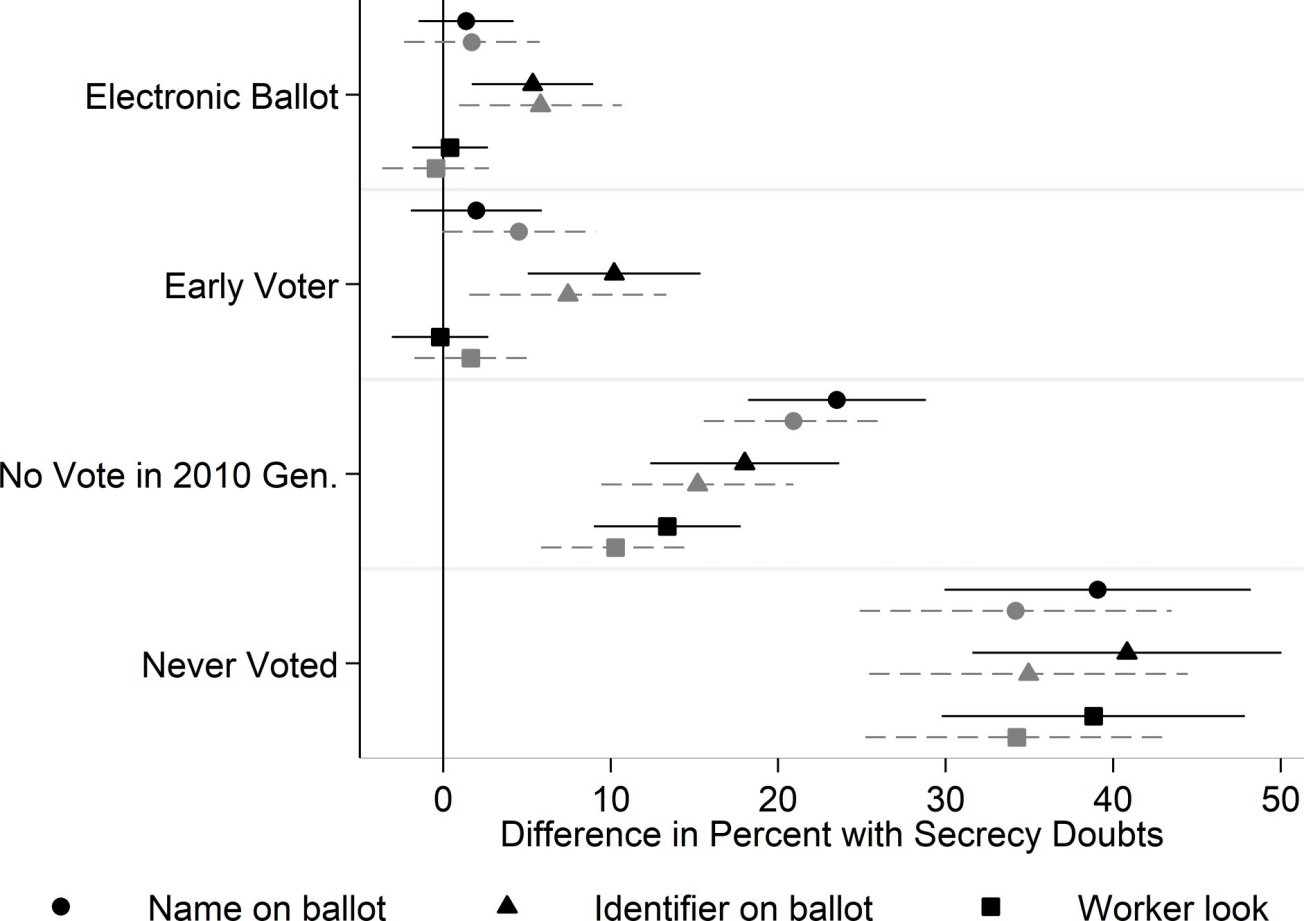
Formal secrecy perceptions. Whiskers indicate 95% confidence intervals. Gray markers are from models that control for respondent" "demographics. Reference categories: Paper ballot; Election Day voter; Voted in 2010 General election.

**Fig 4:**
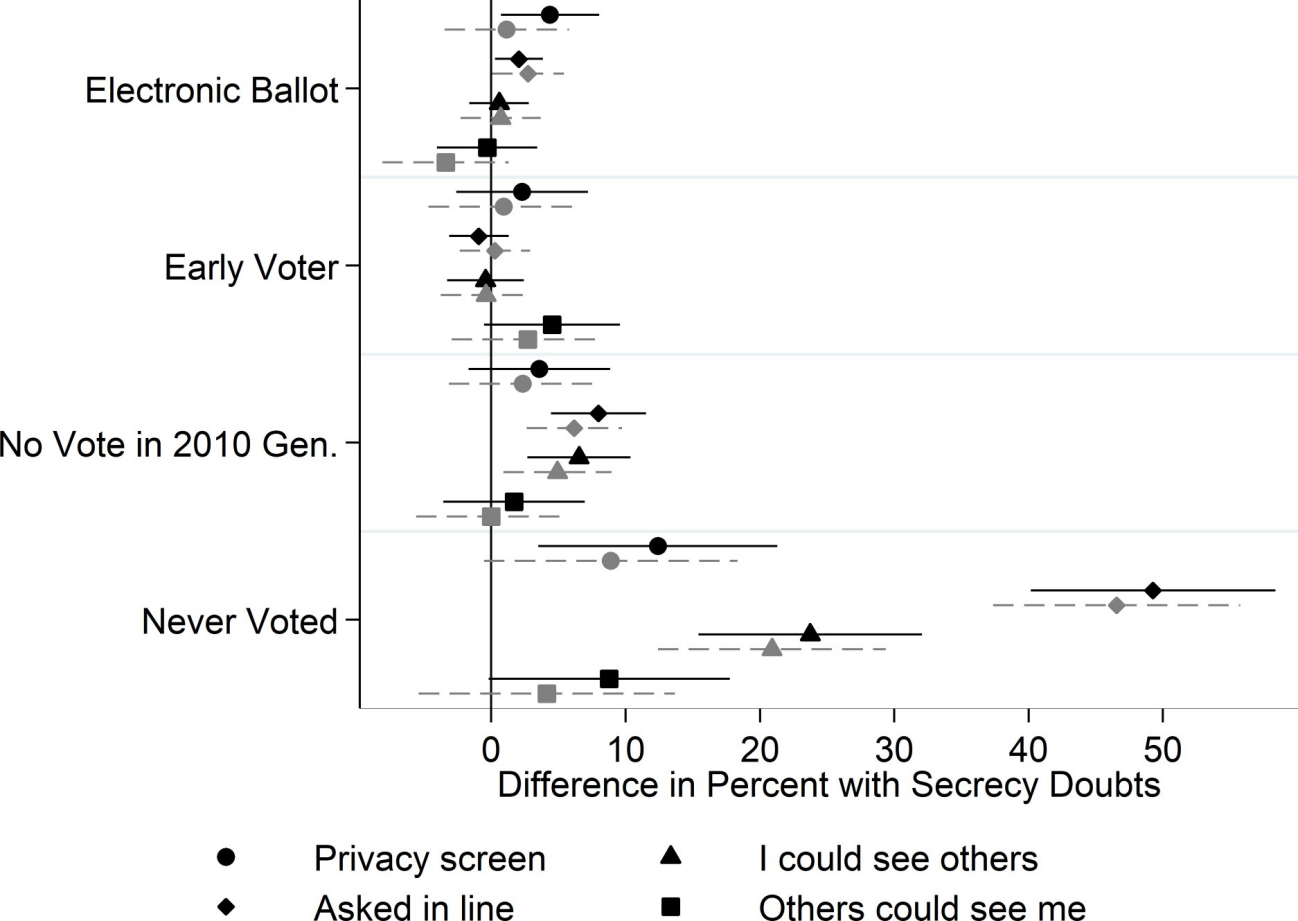
Social secrecy perceptions. Whiskers indicate 95% confidence intervals. Gray markers are from models that control for respondent demographics. Reference categories: Paper ballot; Election Day voter; Voted in 2010 General election.

Next, we assess how secrecy perceptions vary with key features of respondents’ voting experiences. We use OLS regression models to estimate whether secrecy perceptions were tied to voting method and timing (technology, early voting, or voting by mail), or whether the voter had cast a ballot recently (or ever). In each case we set the outcome value to 100 for those who expressed doubts about secrecy (including those who said “don’t know/remember”) and to zero for those who expressed no doubt. We regress each item on indicators for: 1) those who cast electronic ballots the last time they voted (those who cast paper ballots or have never voted are set to zero); 2) early voters and 3) those who voted by mail (each set to zero for non-voters); 4) those who reported having voted but not in the 2010 general election, and 5) those who never voted. Then we estimate a second model controlling for core respondent demographics (race, gender, age, age-squared, education, income, and state of residence) to account for the possibility that any differences of interest may be attributable to a correlation between, say, the decision to vote early and these characteristics (see S3–S5 Tables of the Supplementary information document). As the findings reported in Figs 2–4 illustrate, these controls only modestly affect our estimates of interest.

### Institutional threats to ballot secrecy

We begin by considering responses to the two questions that asked about secrecy in general, as well as the item asking whether elected officials have access to information about the choices voters make when casting a ballot. The coefficients of interest from these models are presented in Fig 2. The findings reported in Fig 2 offer some support for the notion that those who vote electronically harbor greater doubts about whether their choices are secret. Holding other aspects of individuals’ voting experience (e.g., whether they voted early and how recently they voted) constant, those who cast electronic ballots were about four percentage points more likely to say their choices were not secret and that it would be “not at all difficult” for someone to find out who they voted for, even if they told no one about their choices (though the difference for the first item falls short of statistical significance when demographic controls are included; gray markers). These voters were also substantially—about 10 percentage points—more likely than those who cast a paper ballot to say they believed elected officials can access their ballots.

We find no evidence that those who cast ballots early differ systematically from Election Day voters in these top-level perceptions. However, Fig 2 shows a clear pattern where those who reported voting by mail harbor greater doubts about secrecy (compared to those who voted in-person on Election Day). They were almost 10 percentage points more likely to say their choices were not kept secret, and approximately 4 percentage points more likely to say it would be not difficult at all for someone to find out about their choices. They were also 15 percentage points more likely to say they thought elected officials could access their voting records to find out who they voted for. These estimates are largely unchanged when we control for respondent demographics. We return to the experiences of those who reported voting by mail below.

Finally, in Fig 2 we consider the relationship between when an individual last voted and their confidence in the secrecy of the ballot. Fig 2 shows that those who had voted, but did not cast a ballot in the most recent federal election, were more likely to report having doubts about ballot secrecy. Compared to those who voted in the 2010 general election, they were about 4 percentage points more likely to say their choices were not secret and about 13 percentage points more likely to say elected officials could access their choices. Doubts were even more prevalent among non-voters. Compared with those who voted in the last general election, these individuals were 10 and 8 percentage points more likely to express doubts when asked the first two general questions about secrecy. We note that these estimated differences are somewhat imprecise and fall short of conventional thresholds of statistical significance when we control for demographics because our sample includes a fairly small number of respondents who never voted. Those who had never voted were also more than 20 percentage points more likely to say they thought elected officials could access voting records.

Next, we consider differences in experiences and perceptions captured by the three questions that asked more specifically about aspects of the formal voting process that might interfere with secrecy. Before proceeding we note two important features of this analysis. First, respondents who voted by mail were not asked these questions because they pertain to the experience of casting an in-person ballot. Thus, these individuals are excluded from this analysis. Second, respondents who indicated they had never voted were asked analogous questions about what they imagined their voting experiences would be like if they were to vote in person. These questions mirrored those asked of in-person voters except that we asked the questions in the conditional past tense (“would you write your name on your ballot” versus “did you write your name on your ballot”).

Fig 3 reports coefficients from OLS regressions analogous to those used to generate Fig 2. We find little evidence that those who cast electronic (rather than paper) ballots or voted early (rather than on Election Day) were more likely to say they wrote their name on their ballot or that a poll worker looked at their choices. However, these voters were significantly more likely than their counterparts to say their ballot included a code or other piece of personally identifying information.

We find far more pronounced differences tied to how recently respondents’ voted. For example, those who reported having last voted prior to the previous federal election were about 20 percentage points more likely than those who voted in the last election to say they wrote their name on their ballot (or couldn’t remember whether they did). The corresponding difference for recalling whether their ballot included a personal identifier was 15 percentage points. These voters were also notably more likely to say a poll worker had looked at their completed ballot. Those who had never voted expressed even less confidence that their ballots were anonymous and that poll workers would not look at their choices. These individuals were 35 to 40 percentage points more likely to express doubts about these matters.

### Social threats to ballot secrecy

Finally, in Fig 4 we report models tied to the four questions that pertained to whether voters believed their choices were likely to be seen by other voters (reverse coding the question about a privacy screen/curtain so 100 = no screen/don’t know; 0 = there was a screen or curtain). We find little evidence that those who reported voting early differed in their perceptions or experiences from those who voted on Election Day. Similarly, we find little difference between those who cast electronic ballots and those who cast paper ballots.

We do find evidence that those who had voted before but sat out the prior election were more likely to voice doubts about this type of secrecy. Specifically, they were more likely than recent voters to say someone in line asked who they were going to vote for (or that they could not remember). They were also less likely to affirm that they could not see other voters’ ballots. As with the findings reported in Figs 2 and 3, those who never voted were substantially more likely to voice doubts in this domain. They were more likely to have doubts about whether their choices would be shielded by a privacy screen. The largest differences is that those who had never voted were far more likely to voice doubts about whether other voters would ask them who they planned to vote for in line. This finding suggests that those who do not vote may be dissuaded from doing so because they think they may be asked about their choices or asked to justify them while waiting in line. Finally, they were less likely to say they would not be able to see other people’s ballots, but did not differ significantly in their expectations regarding whether others would be able to see their ballot as they filled it out.

We did not ask those who reported voting by mail the same questions as we asked in-person voters. However, there is reason to suspect that the experience of voting is a social one for those who cast votes by mail. For example, we asked these voters whether anyone else was in the room with them when they cast their ballot and over 23 percent of respondents said that at least one other person was in the room with them when they filled out their ballot. We asked those who reported that at least one other person was in the room with them when they filled out their absentee/mail ballot a series of additional questions about their experiences to determine the extent to which casting mail and absentee ballots is a social rather than private experience. Of these respondents, 85 percent said that one person was in the room with them and 14 percent said two or three people were in the room with them. Additionally, 17.2 percent of all respondents who voted by mail said that at least one other person in the room with them while they filled out their ballot was also filling out a ballot, 17.8 percent said they discussed who they were voting for with someone else in the room, and 8.5 percent said they asked someone else for advice about how to vote on a particular race or issue while filling out their ballot. More than six percent of our mail voters reported showing their ballot to someone else after filling it out. In short, for some people the process of casting a mail or absentee ballot is a social act where they reveal their choices to others.

## Discussion

The secret ballot is an important feature of legitimate democratic states. For a voting process to generate legitimacy, however, citizens must be aware of the institutions that are set up to protect the secrecy of the ballot and believe that these institutions are implemented honestly and effectively. The data presented in this paper, however, call into question the assumption that the adoption of formal rules for ballot secrecy in the contemporary United States has fully addressed concerns about the legitimacy and sanctity of the voting process.

For example, many people believe that elected officials can and do access information about who a citizen voted for. Additionally, respondents believe the operation of polling places is insufficient to protect the privacy of choices from election administrators or other citizens. For some of the items we asked in-person voters, as many as 35 percent express explicit doubts about the rules and practices that should be in place to protect ballot secrecy or report experiences that suggest that those protections do not work effectively. While the average respondent believes their ballot is secret, the evidence we present implies that many Americans have at least some doubts about the integrity of the voting process. Even for experienced voters, these doubts may discourage them from coming to the polls or supporting their preferred candidate in some contests.

Additionally, these concerns about ballot secrecy are substantially more common among those who have never previously voted. For example, half of those who had not previously voted think elected officials access voting records and nearly 60 percent either think that there would be personally identifying information on their ballot or do not know. These doubts among those who have not voted before raise a troubling concern: might some citizens decide to stay away from the polls because they anticipate that their choices may be revealed and expose them to unwanted social pressures or sanctions?

At the same time, our evidence also raises doubts about whether changes in voting practices are the best way to remedy these doubts. We find only modest differences between early and Election Day voters and between those casting electronic, rather than paper ballots. Nor does voting by mail, which allows voters to remove themselves from the physical and social interactions at the polling place that might be seen as most obviously threatening ballot secrecy, seem to be a panacea.

Most by-mail voters do not fill out their mail ballots in private and many openly discuss their choices with others at the time that they make their choices. While when a by-mail voter fills out her ballot is under more control than for those who must vote in a polling place, sizeable proportions of those casting ballots by mail report that elected officials can access their voting records after the fact (42 percent). For this reason, it does not appear that errors in the implementation of polling place elections are the sole source of doubts about the integrity of the voting process. Doubts about the institution of the secret ballot may not be resolved with better implementation at the polling place, nor with a move to all-mail elections.

Instead, our results suggest widespread misperceptions about the voting process and ballot secrecy, rather than failures of implementation. Existing research shows that providing information about the voting process from a Secretary of State's office increases turnout [1]. The survey evidence in this paper suggests that the mechanism of this increase in turnout is information that remediates misperception or uncertainty about how voting will be experienced. It may therefore be that effective reforms to increase participation focus on information and familiarity with the voting process.

There are of course some important caveats associated with the results here. First, our analysis is based on self-reports of perceptions and doubts. The behavioral reality and validity of these reports are of crucial importance to the policy implications of our findings, and merit future work on behavioral consequences. The self-reported behaviors and perceptions we rely on may be contaminated by social desirability biases. Reassuringly, the patterns we identify do not change substantially when we use validated rather than self-reported measures of turnout. We have also attempted to characterize the sources of misperceptions and doubts from these survey data, but these are only valid to the extent we have properly accounted for confounding factors and the possibility of reverse causality. For example, one possibility is that individuals who are not disposed to participate offer pessimistic responses to questions about ballot secrecy as a way of justifying their decision to abstain. Future research could consider panel analysis in consideration of this question, however there is also field experimental evidence that messages designed to allay concerns about ballot secrecy increase turnout among those who have previously not voted [1]. This suggests that changing beliefs about ballot secrecy has a causal effect on decisions to vote, consistent with causality operating from beliefs to behavior.

## Supporting information

S1 Appendix Sampling methodology and question wording. (DOCX)

S1 Table Sample characteristics. (DOCX)

S2 Table Distribution of responses to items reported in Fig 1. (DOCX)

S3 Table Top-level secrecy regression models used to generate estimates reported in Fig 2. (DOCX)

S4 Table Formal secrecy protection regression models used to generate estimates reported in Fig 3. (DOCX)

S5 Table Other voters and ballot secrecy regression models used to generate estimates reported in Fig 4. (DOCX)
