# Supplementary material for: The voting experience and beliefs about ballot secrecy
Source: PLoS One. 2019 Jan 7;14(1):e0209765. doi: 10.1371/journal.pone.0209765 (PMC6322754; doi:10.1371/journal.pone.0209765)
Supplement: S1 Appendix — (DOCX) [file pone.0209765.s001.docx]

**S1 Appendix**

**Section A: Sampling Methodology**

Polimetrix/YouGov completed a survey of a nationally representative sample of 3,000 citizens 25-years and older in the month following the November 2010 election on our behalf. This survey data are from an opt-in, internet-based survey. The within-panel participation rate (RR1) was 40.6 percent—3,507 completed interviews out of 8,632 invitations requesting participation (see AAPOR Task Force 2010; there were 148 partial completes, 46 deemed ineligible, and 4,931 who did not respond.). YouGov/Polimetrix uses a combination of sampling and matching techniques to approximate a random digit dialing sample. The final weighted sample (N = 3,000) is nationally representative of the U.S. adult population (age 25 and over).

More specifically, we asked YouGov to interview respondents who had taken both waves of the 2010 Cooperative Congressional Election Study (CCES). The 3,507 completed interviews were then matched on gender, age, race, education, party identification, ideology, and political interest down to a sample of 3,000 to produce the final dataset. YouGov then weighted the matched set of survey respondents to known marginals for the citizen population of the United States age 25+ from the 2006 American Community Survey (ACS). The original CCES sample was constructed by first drawing a target population sample. This sample is based on the 2006 ACS, November 2008 Current Population Survey Supplement, and the 2007 Pew Religious Life Survey. Thus, this target sample is representative of the general population on a broad range of characteristics including a variety of geographic (state, region, metropolitan statistical area), demographic (age, race, income, education, gender), and other measures (born-again status, employment, interest in news, party identification, ideology, and turnout). A stratified sample of individuals from Polimetrix’s opt-in panel was invited to participate in the study. Those who completed the survey were then matched to the target sample based on the variables listed in parentheses above.

**Section B: Question Wording**

- Top-level secrecy perceptions
  - When you cast a ballot, are your candidate and other vote choices kept secret unless you tell someone, or might your ballot be revealed to others or matched to your name without your permission? (100 = Revealed or matched without my permission; 0 = Kept secret)
  - According to the law, which candidate you vote for is supposed to be kept secret unless you tell someone. How difficult do you think it would be for someone to find out who you voted for, even if you told no one? (100 = Not difficult at all; 0 = Not too difficult; 0 = Somewhat difficult; 0 =Very difficult; 0 = Impossible
  - Do you think elected officials access your voting records to figure out who you voted for? (100 = Yes; 0 = No)
- Formal secrecy perceptions *(Note: respondents who reported never having voted were asked question as hypothetical)*
  - Again, thinking about [the last time you voted/if you were to vote]… did you write your name on your ballot? (100 = Yes; 100 = Don’t remember/Don’t know; 0 = No)
  - Again, thinking about [the last time you voted/if you were to vote]… was there any information on your ballot that could be used to identify the ballot as yours? (100 = Yes; 100 = Don’t remember/Don’t know; 0 = No)
  - Again, thinking about [the last time you voted/if you were to vote]… did a poll worker look at your ballot and see who you voted for? (100 = Yes; 100 = Don’t remember/Don’t know; 0 = No)
- Social secrecy perceptions *(Note: respondents who reported never having voted were asked question as hypothetical)*
  - Again, thinking about [the last time you voted/if you were to vote]… was there a curtain or other privacy screen around the place where you filled out your ballot? (0 = Yes; 100 = Don’t remember/Don’t know; 100 = No)
  - Again, thinking about [the last time you voted/if you were to vote]… when you were filling out your ballot, were you able to see who anyone else was voting for? (100 = Yes; 100 = Don’t remember/Don’t know; 0 = No)
  - Again, thinking about [the last time you voted/if you were to vote]… could someone walking by see who you voted for? (100 = Yes; 100 = Don’t remember/DK; 0 = No)
  - Again, thinking about [the last time you voted/if you were to vote]… did someone in line at the polling place ask you who you were voting for? (100 = Yes; 100 = Don’t remember/Don’t know; 0 = No
