# Supplementary material for: The voting experience and beliefs about ballot secrecy
Source: PLoS One. 2019 Jan 7;14(1):e0209765. doi: 10.1371/journal.pone.0209765 (PMC6322754; doi:10.1371/journal.pone.0209765)
Supplement: S1 Table — (DOCX) [file pone.0209765.s002.docx]

| Variable | (1) |
| --- | --- |
| Electronic Ballot | 0.382 |
|  | [.486] |
| Early Voter | 0.135 |
|  | [.3421] |
| Vote by Mail | 0.191 |
|  | [.3928] |
| No Vote in 2010 Gen. | 0.129 |
|  | [.3349] |
| Never Voted | 0.042 |
|  | [.2008] |
| Race: Black (1=yes) | 0.109 |
|  | [.3119] |
| Race: Hispanic (1=yes) | 0.069 |
|  | [.2532] |
| Race: Other Race (1=yes) | 0.060 |
|  | [.2378] |
| Female (1=yes) | 0.521 |
|  | [.4996] |
| Age (Years) | 50.498 |
|  | [13.8847] |
| Age-squared/100 | 27.428 |
|  | [14.3626] |
| Education (1=No HS; 6=Post-grad) | 3.348 |
|  | [1.4557] |
| Income (1=<10k; 14=>150k; 15=RF/Skipped) | 8.401 |
|  | [3.9313] |
| Income Missing | 0.092 |
|  | [.2888] |
| Observations | 2876 |
| Values are *unweighted* means. Standard deviations in brackets | |
