# Supplementary material for: The voting experience and beliefs about ballot secrecy
Source: PLoS One. 2019 Jan 7;14(1):e0209765. doi: 10.1371/journal.pone.0209765 (PMC6322754; doi:10.1371/journal.pone.0209765)
Supplement: S2 Table — (DOCX) [file pone.0209765.s003.docx]

|  | Reported Voting in Person in Last Election (Figure 1) | | | | Reported Voting in Person in Last Election + Validated as Voting in 2010 | | | | Differences | | |
| --- | --- | --- | --- | --- | --- | --- | --- | --- | --- | --- | --- |
|  | % Yes | % Don't Remember | % No | N | % Yes | % Don't Remember | % No | N | % Yes | % Don't Remember | % No |
| …thinking about the last time you voted, did you write your name on your ballot? | 5.7% | 7.0% | 87.4% | 2198 | 3.5% | 3.4% | 93.1% | 1203 | 2.2% | 3.6% | -5.7% |
| …thinking about the last time you voted, was there any information on your ballot that could be used to identify the ballot as yours? | 11.1% | 12.8% | 76.1% | 2200 | 9.7% | 8.8% | 81.5% | 1205 | 1.4% | 4.0% | -5.4% |
| …thinking about the last time you voted, did a poll worker look at your ballot and see who you voted for? | 2.6% | 4.7% | 92.8% | 2205 | 1.6% | 2.5% | 95.9% | 1209 | 1.0% | 2.1% | -3.1% |
| …thinking about the last time you voted, was there a curtain or other privacy screen around the place where you filled out your ballot? | 75.6% | 2.2% | 22.2% | 2201 | 75.7% | 0.9% | 23.4% | 1209 | -0.1% | 1.3% | -1.2% |
| …thinking about the last time you voted, when you were filling out your ballot, were you able to see who anyone else was voting for? | 5.2% | 1.8% | 93.0% | 2201 | 4.0% | 0.5% | 95.5% | 1207 | 1.3% | 1.2% | -2.5% |
| …thinking about the last time you voted, could someone walking by see who you voted for? | 21.1% | 5.0% | 73.8% | 2199 | 21.1% | 3.8% | 75.0% | 1206 | 0.0% | 1.2% | -1.2% |
| …thinking about the last time you voted, did someone in line at the polling place ask you who you were voting for? | 2.6% | 1.6% | 95.8% | 2197 | 1.0% | 0.3% | 98.7% | 1208 | 1.6% | 1.2% | -2.9% |
| Note: Cell entries are the percentage of respondents providing each response. The first set of columns are the values reported in Figure 1 of the manuscript. The next set of columns restricts the sample to respondents who were able to be validated as having voted in the 2010 general election. The remaining columns report the differences across these sample restrictions. Consistent with findings reported elsewhere in the article, those validated as having voted in the most recent election were modestly less likely to provide responses that indicated doubts about ballot secrecy. | | | | | | | | | | | |
