# Supplementary material for: The voting experience and beliefs about ballot secrecy
Source: PLoS One. 2019 Jan 7;14(1):e0209765. doi: 10.1371/journal.pone.0209765 (PMC6322754; doi:10.1371/journal.pone.0209765)
Supplement: S3 Table — (DOCX) [file pone.0209765.s004.docx]

|  | (1) | (2) | (3) | (4) | (5) | (6) |
| --- | --- | --- | --- | --- | --- | --- |
|  | ...are your candidate and other vote choices kept secret unless you tell someone, or might your ballot be revealed to others or matched to your name without your permission?  (100 = Can be revealed; 0 = Kept secret) | | ...How difficult do you think it would be for someone to find out who you voted for, even if you told no one? (100 = Not too difficult at all; 0 = Not too difficult - Impossible) | | Do you think elected officials access your voting records to figure out who you voted for?  (100 = Yes; 0 = No) | |
| Electronic Ballot | 4.391 | 1.958 | 4.004 | 4.100 | 0.132 | 0.099 |
|  | [1.360]** | [1.661] | [1.375]** | [1.711]* | [0.033]** | [0.042]* |
| Early Voter | 3.056 | 2.901 | 0.178 | 0.782 | 0.025 | -0.005 |
|  | [1.953] | [2.093] | [1.837] | [1.939] | [0.045] | [0.052] |
| Vote by Mail | 9.924 | 9.466 | 3.548 | 3.660 | 0.151 | 0.181 |
|  | [1.853]** | [2.163]** | [1.696]* | [1.993] | [0.040]** | [0.048]** |
| No Vote in 2010 Gen. | 4.693 | 3.555 | 0.986 | -0.185 | 0.139 | 0.131 |
|  | [2.021]* | [2.095] | [1.867] | [1.941] | [0.047]** | [0.048]** |
| Never Voted | 10.606 | 7.287 | 8.038 | 6.252 | 0.233 | 0.212 |
|  | [3.587]** | [3.678]* | [3.581]* | [3.624] | [0.075]** | [0.079]** |
| Race: Black (1=yes) |  | 1.566 |  | 3.406 |  | 0.077 |
|  |  | [2.285] |  | [2.255] |  | [0.050] |
| Race: Hispanic (1=yes) |  | 1.073 |  | 4.109 |  | 0.021 |
|  |  | [2.601] |  | [2.769] |  | [0.062] |
| Race: Other Race (1=yes) |  | 5.096 |  | 2.317 |  | 0.074 |
|  |  | [3.082] |  | [2.723] |  | [0.060] |
| Female (1=yes) |  | -2.539 |  | -0.249 |  | -0.087 |
|  |  | [1.275]* |  | [1.242] |  | [0.030]** |
| Age (Years) |  | 0.638 |  | 0.940 |  | 0.014 |
|  |  | [0.293]* |  | [0.265]** |  | [0.007] |
| Age-squared/100 |  | -0.736 |  | -0.952 |  | -0.013 |
|  |  | [0.277]** |  | [0.249]** |  | [0.007] |
| Education (1=No HS; 6=Post-grad) |  | -0.274 |  | -1.061 |  | -0.034 |
|  |  | [0.467] |  | [0.445]* |  | [0.011]** |
| Income (1=<10k; 14=>150k; 15=RF/Skipped) |  | -0.479 |  | -0.397 |  | -0.007 |
|  |  | [0.215]* |  | [0.200]* |  | [0.005] |
| Income Missing |  | 3.345 |  | 6.262 |  | 0.118 |
|  |  | [2.595] |  | [2.618]* |  | [0.060] |
| State fixed effects? | No | Yes | No | Yes | No | Yes |
| Constant | 7.178 | -0.509 | 8.840 | -6.880 | 0.265 | -0.001 |
|  | [0.855]** | [7.943] | [0.930]** | [7.332] | [0.023]** | [0.196] |
| Observations | 2861 | 2861 | 2869 | 2869 | 1130 | 1130 |
| R-squared | 0.015 | 0.038 | 0.005 | 0.034 | 0.032 | 0.112 |
| Note: Cell entries are unstandardized OLS coefficients from regression models using sample weights. Robust standard errors in brackets. Outcome variable used in columns (5) and (6) asked of random subsample of respondents. * significant at 5%; ** significant at 1%. | | | | | | |
