# Supplementary material for: The voting experience and beliefs about ballot secrecy
Source: PLoS One. 2019 Jan 7;14(1):e0209765. doi: 10.1371/journal.pone.0209765 (PMC6322754; doi:10.1371/journal.pone.0209765)
Supplement: S4 Table — (DOCX) [file pone.0209765.s005.docx]

|  | (1) | (2) | (3) | (4) | (5) | (6) |
| --- | --- | --- | --- | --- | --- | --- |
|  | …write your name on your ballot? | | ...any information on your ballot that could be used to identify the ballot as yours? | | ...poll worker look at your ballot and see who you voted for? | |
|  | Yes or DK/don't remember = 1; No = 0 | | | | | |
| Electronic Ballot | 0.014 | 0.017 | 0.053 | 0.058 | 0.004 | -0.004 |
|  | [0.015] | [0.021] | [0.018]** | [0.025]* | [0.012] | [0.016] |
| Early Voter | 0.020 | 0.045 | 0.102 | 0.074 | -0.002 | 0.016 |
|  | [0.020] | [0.023] | [0.026]** | [0.030]* | [0.015] | [0.017] |
| No Vote in 2010 Gen. | 0.235 | 0.209 | 0.180 | 0.152 | 0.134 | 0.103 |
|  | [0.027]** | [0.027]** | [0.029]** | [0.029]** | [0.022]** | [0.023]** |
| Never Voted | 0.391 | 0.342 | 0.408 | 0.349 | 0.388 | 0.342 |
|  | [0.047]** | [0.047]** | [0.047]** | [0.048]** | [0.046]** | [0.046]** |
| Race: Black (1=yes) |  | 0.078 |  | 0.061 |  | 0.057 |
|  |  | [0.026]** |  | [0.030]* |  | [0.023]* |
| Race: Hispanic (1=yes) |  | 0.038 |  | 0.051 |  | -0.005 |
|  |  | [0.035] |  | [0.039] |  | [0.028] |
| Race: Other Race (1=yes) |  | 0.007 |  | -0.003 |  | 0.027 |
|  |  | [0.030] |  | [0.039] |  | [0.027] |
| Female (1=yes) |  | 0.012 |  | -0.005 |  | 0.024 |
|  |  | [0.014] |  | [0.018] |  | [0.012]* |
| Age (Years) |  | -0.001 |  | 0.000 |  | -0.003 |
|  |  | [0.004] |  | [0.004] |  | [0.003] |
| Age-squared/100 |  | -0.001 |  | -0.002 |  | 0.001 |
|  |  | [0.003] |  | [0.004] |  | [0.003] |
| Education (1=No HS; 6=Post-grad) |  | -0.018 |  | -0.023 |  | -0.008 |
|  |  | [0.005]** |  | [0.006]** |  | [0.004]* |
| Income (1=<10k; 14=>150k; 15=RF/Skipped) |  | -0.006 |  | -0.003 |  | -0.004 |
|  |  | [0.002]* |  | [0.003] |  | [0.002] |
| Income Missing |  | 0.043 |  | 0.043 |  | 0.018 |
|  |  | [0.028] |  | [0.036] |  | [0.022] |
| State fixed effects? | No | Yes | No | Yes | No | Yes |
| Constant | 0.078 | 0.259 | 0.166 | 0.321 | 0.049 | 0.239 |
|  | [0.009]** | [0.099]** | [0.012]** | [0.121]** | [0.008]** | [0.083]** |
| Observations | 2319 | 2319 | 2320 | 2320 | 2326 | 2326 |
| R-squared | 0.108 | 0.159 | 0.066 | 0.131 | 0.112 | 0.158 |
| Note: Cell entries are unstandardized OLS coefficients from regression models using sample weights. Robust standard errors in brackets. * significant at 5%; ** significant at 1%. | | | | | | |
