# Supplementary material for: The voting experience and beliefs about ballot secrecy
Source: PLoS One. 2019 Jan 7;14(1):e0209765. doi: 10.1371/journal.pone.0209765 (PMC6322754; doi:10.1371/journal.pone.0209765)
Supplement: S5 Table — (DOCX) [file pone.0209765.s006.docx]

|  | (1) | (2) | (3) | (4) | (5) | (6) | (7) | (8) |
| --- | --- | --- | --- | --- | --- | --- | --- | --- |
|  | ...curtain or other privacy screen around the place where you filled out your ballot? | | ...able to see who anyone else was voting for? | | ...someone walking by see who you voted for? | | ...someone in line at the polling place ask you who you were voting for? | |
|  | Yes or DK/don't remember = 1; No = 0 | | | | | | | |
| Electronic Ballot | 0.044 | 0.012 | 0.006 | 0.007 | -0.003 | -0.034 | 0.021 | 0.027 |
|  | [0.019]* | [0.024] | [0.011] | [0.015] | [0.019] | [0.024] | [0.009]* | [0.014]* |
| Early Voter | 0.023 | 0.009 | -0.004 | -0.004 | 0.045 | 0.027 | -0.009 | 0.003 |
|  | [0.025] | [0.029] | [0.015] | [0.017] | [0.026] | [0.029] | [0.011] | [0.013] |
| No Vote in 2010 Gen. | 0.036 | 0.024 | 0.065 | 0.049 | 0.017 | 0.000 | 0.080 | 0.062 |
|  | [0.027] | [0.028] | [0.020]** | [0.020]* | [0.027] | [0.028] | [0.018]** | [0.018]** |
| Never Voted | 0.124 | 0.089 | 0.237 | 0.209 | 0.088 | 0.041 | 0.493 | 0.466 |
|  | [0.045]** | [0.048] | [0.042]** | [0.043]** | [0.046] | [0.049] | [0.046]** | [0.047]** |
| Race: Black (1=yes) |  | 0.006 |  | 0.053 |  | -0.011 |  | 0.040 |
|  |  | [0.030] |  | [0.022]* |  | [0.031] |  | [0.020]* |
| Race: Hispanic (1=yes) |  | -0.016 |  | 0.027 |  | -0.027 |  | 0.004 |
|  |  | [0.039] |  | [0.029] |  | [0.040] |  | [0.024] |
| Race: Other Race (1=yes) |  | 0.000 |  | 0.020 |  | -0.039 |  | 0.049 |
|  |  | [0.040] |  | [0.027] |  | [0.041] |  | [0.025]* |
| Female (1=yes) |  | 0.008 |  | 0.014 |  | 0.025 |  | 0.021 |
|  |  | [0.018] |  | [0.012] |  | [0.019] |  | [0.010]* |
| Age (Years) |  | 0.003 |  | 0.001 |  | 0.007 |  | -0.003 |
|  |  | [0.005] |  | [0.003] |  | [0.004] |  | [0.003] |
| Age-squared/100 |  | -0.004 |  | -0.003 |  | -0.011 |  | 0.001 |
|  |  | [0.004] |  | [0.003] |  | [0.004]** |  | [0.002] |
| Education (1=No HS; 6=Post-grad) |  | 0.009 |  | -0.003 |  | 0.013 |  | -0.006 |
|  |  | [0.007] |  | [0.004] |  | [0.007] |  | [0.003] |
| Income (1=<10k; 14=>150k; 15=RF/Skipped) |  | -0.002 |  | 0.001 |  | -0.001 |  | -0.002 |
|  |  | [0.003] |  | [0.002] |  | [0.003] |  | [0.002] |
| Income Missing |  | 0.021 |  | -0.017 |  | 0.038 |  | 0.019 |
|  |  | [0.039] |  | [0.023] |  | [0.039] |  | [0.018] |
| State fixed effects? | No | Yes | No | Yes | No | Yes | No | Yes |
| Constant | 0.212 | 0.185 | 0.058 | 0.046 | 0.253 | 0.118 | 0.021 | 0.154 |
|  | [0.013]** | [0.120] | [0.008]** | [0.079] | [0.014]** | [0.121] | [0.005]** | [0.071]* |
| Observations | 2320 | 2320 | 2322 | 2322 | 2320 | 2320 | 2316 | 2316 |
| R-squared | 0.007 | 0.054 | 0.043 | 0.081 | 0.003 | 0.064 | 0.200 | 0.231 |
| Note: Cell entries are unstandardized OLS coefficients from regression models using sample weights. Robust standard errors in brackets. * significant at 5%; ** significant at 1%. | | | | | | | | |
